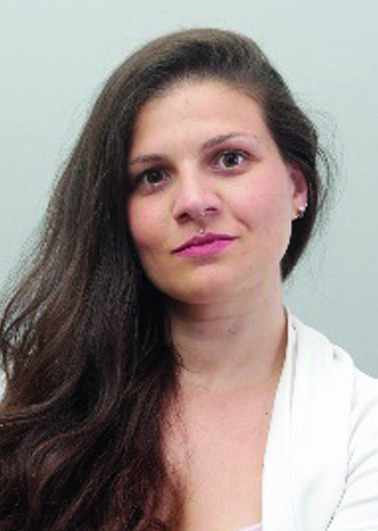# Cracking Lysine Crotonylation (Kcr): Enlightening a Promising Post‐Translational Modification

**DOI:** 10.1002/cbic.202400639

**Published:** 2024-10-27

**Authors:** Marinda Westerveld, Kosta Besermenji, David Aidukas, Nikita Ostrovitsa, Rita Petracca

**Affiliations:** ^1^ Department of Pharmaceutical Sciences Faculty of Science Utrecht University David De Wied Building, Universiteitsweg 99 3584 CG Utrecht NL; ^2^ Trinity Biomedical Sciences Institute (TBSI) Trinity College Dublin (TCD) 152-160 Pearse St. Dublin D02 R590 Ireland

**Keywords:** Post-translational modifications (PTMs), Crotonyl-CoA, Enzymatic PTMs regulation, Chemical proteomics

## Abstract

Lysine crotonylation (Kcr) is a recently discovered post‐translational modification (PTM). Both histone and non‐histone Kcr‐proteins have been associated with numerous diseases including cancer, acute kidney injury, HIV latency, and cardiovascular disease. Histone Kcr enhances gene expression to a larger extend than the extensively studied lysine acetylation (Kac), suggesting Kcr as a novel potential therapeutic target. Although numerous scientific reports on crotonylation were published in the last years, relevant knowledge gaps concerning this PTM and its regulation still remain. To date, only few selective Kcr‐interacting proteins have been identified and selective methods for the enrichment of Kcr‐proteins in chemical proteomics analysis are still lacking. The development of new techniques to study this underexplored PTM could then clarify its function in health and disease and hopefully accelerate the development of new therapeutics for Kcr‐related disease. Herein we briefly review what is known about the regulation mechanisms of Kcr and the current methods used to identify Kcr‐proteins and their interacting partners. This report aims to highlight the significant potential of Kcr as a therapeutic target and to identify the existing scientific gaps that new research must address.

## Graphical abstract

Lysine crotonylation (Kcr) is a novel post‐translational modification linked to diseases like cancer and cardiovascular disease. Kcr enhances gene expression more than lysine acetylation, making it a promising therapeutic target. Despite recent research, key regulatory mechanisms and selective methods for studying Kcr remain underexplored. This review highlights Kcr's potential and identifies gaps needing further investigation.

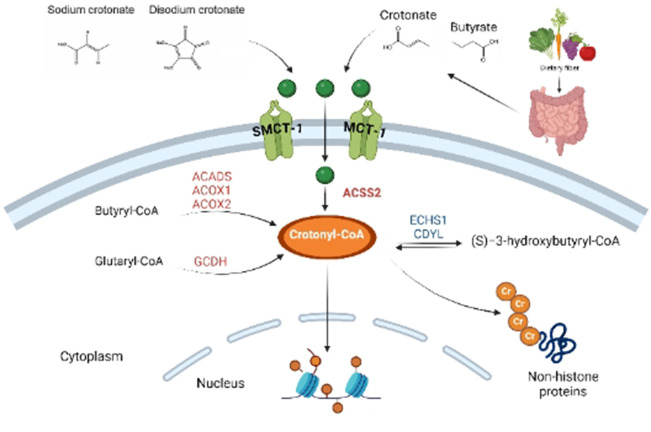

## 1 Introduction

Protein post‐translational modifications (PTMs) are important players in protein folding, activity, stability, localization, and the interaction with other cellular entities.[^1^, ^2^] As suggested by the name, PTMs modify the target protein after translation and they are responsible for the high complexity of cellular proteomes.^[3]^

Chemically, PTMs correspond to different molecules attached covalently to specific amino acid lateral chains; to date, 400 different types of PTMs have been identified.^[4]^ Dysregulation of PTMs is associated with numerous diseases, including cancer, neuropsychiatric disease, tissue injury, and cardiovascular disease.^[5]^ Among the most studied PTMs, lysine acetylation (Kac) was described as the first lysine acylation in the 1960s.^[6]^ Since its discovery, many researchers have focused on investigating this PTM and its specific role in health and disease.[^7^, ^8^, ^9^] This extensive investigation led, after approximately 50 years, to the development of several FDA approved drugs against Kac, such as Vorinostat (2006) and Belinostat (2014) to treat cutaneous T cell lymphoma, and Selisistat (2009) for the treatment of multiple myeloma.^[7]^

**Figure 1:**
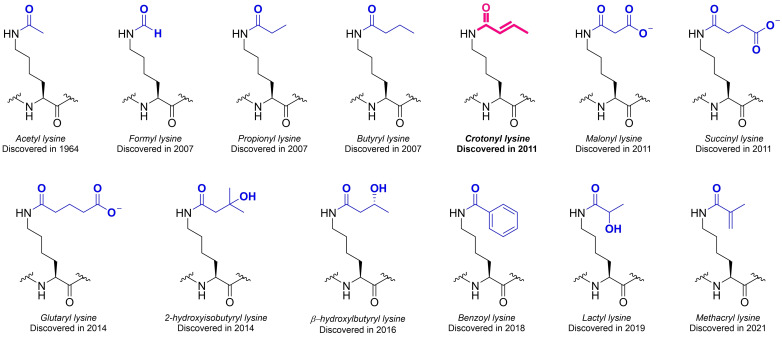
Short lysine acylations and their discovery year: acetylation,^[6]^ formylation,[^11^, ^12^] propionylation,^[13]^ butyrylation,^[13]^ crotonylation,^[14]^ malonylation,^[15]^ succinylation,^[16]^ glutarylation,^[17]^ 2‐hydroxyisobutyrylation,^[18]^ β‐hydroxybutyrylation,^[19]^ benzoylation,^[20]^ lactylation,^[21]^ methacrylation.^[22]^

High‐resolution liquid chromatography coupled to tandem mass spectrometry (LC‐MS/MS) is the gold standard for the investigation and the identification of protein modifications;^[10]^ significant recent advancements in the proteomic and metabolomic fields allowed for the discovery of novel PTMs. Particularly, short‐chain (SC) lysine acylations are exponentially attracting the scientists’ attention (Figures 1[^6^, ^11^, ^12^, ^13^, ^14^, ^15^, ^16^, ^17^, ^18^, ^19^, ^20^, ^21^, ^22^]).[^23^, ^24^, ^25^, ^26^, ^27^] SC K‐acylation seems to be highly dependent on the environmental conditions and metabolic changes,^[28]^ suggesting their fundamental role in the complex cellular metabolic reprogramming, often associated with disease.[^29^, ^30^, ^31^, ^32^]

By all of the SC K‐acylations, lysine crotonylation (Kcr) stands out as an interesting but still understudied PTM.^[14]^ Similarly to the other lysine acylations, Kcr occurs on the ϵ‐amino group of lysine, where it neutralizes the positive charge of the residue^[33]^; the Kcr group is transferred to the substrate protein in the form of crotonyl‐coenzyme A (crotonyl‐CoA).^[34]^ The levels of Kcr on proteins are regulated both by the intracellular levels of crotonyl‐CoA,^[35]^ and by the enzymatic activity of specific proteins able to recognise (*readers*), append (*writers*), and remove (*erasers*) this protein modification. Structurally, Kcr contains an additional C−C π‐bond compared to Kac, giving the crotonyl group a more rigid and planar structure, which makes it unique also compared to other lysine acyl‐groups (Figure 1).

### 1.1 Kcr Mechanisms in Gene Expression

Kcr was first identified by Tan *et al*. in 2011, when they aimed to identify novel histone PTM sites using an integrated MS‐based proteomics approach.^[14]^ They identified a total of 28 Kcr sites on the tails of the core histones H2A, H2B, H3, and H3. The authors demonstrated that histone crotonylation specifically marked the enhancers and transcription start sites of active genes in human somatic and mouse male germ cell genomes, suggesting a relationship between crotonylation and gene activation.^[14]^ The mechanism by which histone Kcr activates gene transcription was later revealed by Suzuki *et al*. when they obtained the crystal structure of the nucleosome containing crotonylated H3K122cr.^[36]^ They showed that crotonylation does not affect the overall structure of the nucleosome, but hinders the formation of water‐mediated hydrogen bonds with the DNA backbone.^[36]^ This weakens the local histone‐DNA interactions, resulting in a less compact chromatin structure, making the DNA more accessible to DNA binding factors, which ultimately leads to transcriptional activation. On a mechanistic level, Kcr influences gene expression in a similar way as Kac, however, gene expression is enhanced to a greater degree by Kcr compared to Kac, due to the increased bulk and rigid nature of the crotonyl group.^[35]^

The link between histone Kcr and gene activation was further confirmed by Montellier *et al*.; in their study, they showed that histone Kcr plays an important role in maintaining X/Y‐linked genes active in post‐meiotic male germ cells, and emphasized that histone Kcr might be an indicator of the male haploid cell gene expression program.^[37]^ In the same year, Sin *et al*. reported histone Kcr as one of multiple modifications that lead to gene activation from inactive sex chromosomes in post‐meiotic spermatids.^[38]^ Moreover, accumulation of Kcr at transcriptional start sites of sex‐linked genes was found to induce a chromatin conformational change in an RNF8‐dependent manner.

The substantial impact that histone Kcr has on health, became even more clear when Ruiz‐Anders *et al*. showed that gene activation as a result of histone crotonylation plays a key role in acute kidney injury (AKI).^[39]^ An increase in kidney tissue histone Kcr was observed during AKI, specifically at the genes encoding peroxisome‐proliferator‐activated receptor gamma coactivator‐1α (PGC‐1α), a mitochondrial biogenesis regulator, and sirtuin‐3 (SIRT3), a decrotonylase. The effect of crotonylation on PGC‐1α and SIRT3 expression was further investigated by incubating tubular cells or healthy kidneys *in vivo* with crotonate, which has previously been shown to increase histone crotonylation.^[14]^ An increase in the expression of PGC‐1α and SIRT3 was observed after crotonate treatment. Additionally, crotonate decreased tubular cell CCL2, which encodes the MCP‐1 chemokine, a promotor of kidney injury. These findings suggest a protective role of histone crotonylation in AKI, by promoting the upregulation of protective genes, while downregulating genes associated with tissue injury, showing the potential of histone crotonylation as therapeutic target.^[39]^

Interestingly, histone crotonylation is not only associated with activation of gene expression but can also decrease gene expression. This down‐regulating effect has been shown by Wang *et al*. in Alzheimer's disease (AD).^[40]^ AD is characterised by accumulation of intracellular amyloid‐beta (Aβ). Wang *et al*. showed that the expression of nuclear paraspeckle assembly transcript 1 (NEAT1) is down‐regulated in early‐stage AD. The decrease in NEAT1 inhibits the expression of endocytosis‐related genes, which in turn decreases neuroglial cell‐mediated Aβ clearance. Mechanistically, NEAT1 colocalizes with the acetyltransferase p300/CREB‐binding protein (CBP) complex and mediates autoacetylation and acetyltransferase activities of p300. Inhibition of NEAT1 was shown to downregulate H3 K27ac, while upregulating H3 K27cr located close to the transcription start site of many genes. Moreover, they showed that NEAT1 mediates the binding between transcriptional factor STAT3 and H3 K27ac, but not with H3 K27cr. Together, resulting in a decrease in the expression of multiple endocytosis‐related genes.^[40]^

**Figure 2:**
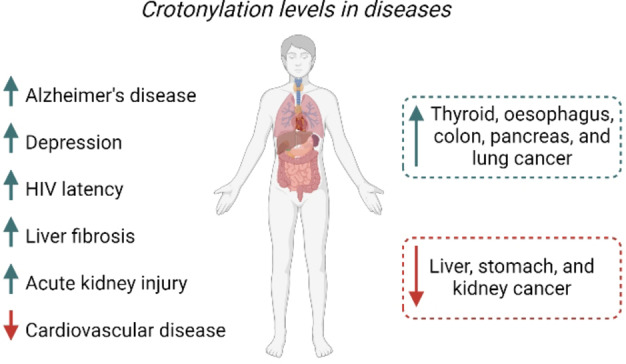
The function of Kcr in disease; increased levels of Kcr are indicated with an upwards arrow and a downwards arrow indicates downregulation of Kcr in the respective disease. The involvement of Kcr in the diseases reported in the dashed squares is only hypothesised, not yet confirmed.

Six years after the discovery of Kcr as a novel histone PTM, Wei *et al*. reported Kcr on non‐histone proteins for the first time.^[41]^ They identified hundreds of crotonylated proteins, using selective antibody enrichment followed by high‐resolution mass spectrometry. Shortly after, Xu *et al*. reported 2696 crotonylation sites on 1024 non‐histone proteins in H1299 cells.^[42]^ These first attempts of describing the *crotonylome* showed that the crotonylated proteins were largely distributed throughout cells, thus participating in a wide variety of important cellular pathways. In fact, since its discovery, it has been shown that crotonylation of non‐histone proteins is responsible for altered protein activity, protein localization, protein degradation, and plays an important role in DNA repair.^[41]^ To date, there are reports demonstrating the involvement of both histone and non‐histone crotonylation in numerous diseases of relevant social impact, such as AKI, AD, depression, HIV latency, cancer, cardiovascular diseases, and liver fibrosis (Figure 2).^[43]^

### 1.2 Kcr in DNA Damage Response

In certain pathways, Kcr exhibits its effect both through histone and non‐histone crotonylation, for example during the DNA damage response (DDR). An important protein during DDR is the replication protein A (RPA), a single‐stranded DNA (ssDNA) binding protein. Binding of RPA prevents ssDNA from forming secondary structures or from winding back on itself, which is an important step during DNA metabolic processes such as replication, repair, and homologous recombination in meiosis.^[44]^ Crotonylation of RPA1 promotes its binding to ssDNA and thereby improves the DNA damage response. Crotonylation of RPA1 is negatively regulated by chromodomain Y‐like (CDYL) protein, which functions as a crotonyl‐CoA hydratase. Yu *et al*. showed that the knockout of CDYL in HeLa cells increases K88cr, K379cr, and K595cr of RPA1, which promoted the DNA damage response.^[45]^

In addition to the effect of CDYL on DDR by decreasing non‐histone crotonylation, CDYL also elucidates an effect on DDR via histone crotonylation. Abu‐Zhayia *et al*. showed that CDYL1 decreases H3K9cr at double‐strand break (DSB) sites, which leads to the eviction of the transcription elongation factor ENL, silencing the genes around the DNA damage site.^[46]^ A common belief is that transcriptional silencing, as a result of DSBs, avoids clashes between repair and transcription factors at DSB sites, thus preventing the formation of incomplete and abnormal transcripts.^[47]^ However, Abu‐Zhayia *et al*. found that while inhibition of CDYL1 blocks the reduction in H3 K9cr and promotes transcriptional silencing at DSB sites, homologous recombination efficiency remains unaffected.^[46]^ This indicates that repair and silencing activity of CDYL1 at DBS sites might functionally be uncoupled, underlining the importance of understanding the mechanism and function of lysine crotonylation.

### 1.3 Kcr in Depression

Not only understanding the interplay between histone and non‐histone crotonylation is of great importance, also the balance between different lysine modifications has a great impact in diseases; for example, in one of the pathways involved in depression, Liu *et al*. found decreased levels of histone Kcr in the medial prefrontal cortex in combination with upregulated CDYL in stress‐susceptible rodents.^[48]^ The increase of CDYL levels led to an increase in the depression phenotype. Knocking down of CDYL in the prelimbic cortex led to an antidepressant response. CDYL exhibits its effects by increasing H3K27me3 and decreasing histone Kcr on the VGF nerve growth factor inducible promoter region, leading to lowered structural synaptic plasticity, ultimately leading to depression‐like behaviour in mice.^[48]^

### 1.4 Kcr in Cancer

Another interesting lysine acylation crosstalk is observed for Kac and Kcr. In gene expression, Kac or Kcr of histones can both lead to activation of gene expression, with Kcr being a stronger enhancer compared to Kcr. Interestingly, Kac and Kcr can also lead to different outcomes. For example, lysine acetylation of tumour suppressor p53 promotes its transcription activity and the expression of proapoptotic genes.[^49^, ^50^] Moreover, Kac promotes p53 stability, by preventing the binding of p53 to its E3 ubiquitin ligase MDM2, ultimately downregulating its proteosome degradation.^[51]^ It has been reported that p53 can also be crotonylated at Ser46.[^41^, ^52^] Surprisingly, the authors found that crotonylation of p53 reduces the amount of p53 only at protein level, not at mRNA level, and that this is MDM2‐ and ubiquitination‐independent.^[52]^ This leads then to an impairment of p53 activity, thus augmenting cancer cell proliferation.

Like Kac, Kcr seems to be involved in various types of cancer. Surprisingly, the expression of Kcr is downregulated in liver, stomach, and kidney cancer, while in thyroid, oesophagus, colon, pancreas, and lung cancer, an upregulation of Kcr levels has been found.^[53]^ The different effects are thought to be a result of the regulation of different key cancer‐related proteins via histone and non‐histone crotonylation in different cancer types. Leading to a rise in complexity, histone and non‐histone crotonylation may even exhibit opposing effects within the same cancer type. In hepatocellular carcinoma (HCC), for example, Wan *et al*. found that Kcr is correlated with Tumour, Node, and Metastasis (TNM) stage. However, downregulation of crotonyl *erasers* and consequent increase of Kcr expression was found to illogically inhibit hepatoma cell migration and proliferation.^[53]^

Another recent study, demonstrated that crotonylation of the GTPase Septin 2 promotes cell invasion and metastasis in HCC cells, leading to poor prognosis and a high recurrence rate in HCC patients.^[54]^ Zhang *et al*. found that in liver cancer protein Kcr is upregulated in response to hypoxia and promotes liver cancer cell proliferation, while it prevents cellular senescence.^[55]^ In this same study, a direct correlation between treatment with sodium crotonate and tumour size *in vivo* was found, highlighting the key role for crotonylation in cancer, potentially forming a novel target for cancer therapies.

Studying more in‐depth the role of protein crotonylation could potentially accelerate the development of treatment options for incurable cancers. Interestingly, analysing the mechanism of action of certain available treatment options and their effect on crotonylation levels, can also give more insights in the role of Kcr. This was the case when Chen *et al*. investigated the mechanism of action of sorafenib in liver fibrosis.^[56]^ They revealed a decrease in total Kcr in a liver fibrosis mouse model, caused by upregulation of the crotonyl *erasers* HDAC1, HDAC3, and CDYL. Sorafenib treatment decreased the levels of these crotonyl *erasers*, restoring the Kcr levels in fibrotic livers, suggesting a link between liver fibrosis and crotonylation.

The here described mechanisms of protein crotonylation are solely a short summary of the role of crotonylation in diseases. The extensive role of crotonylation is becoming clearer every year.[^34^, ^43^, ^57^, ^58^] We can safely say that there is enough evidence showing how Kcr could be an interesting therapeutic target; question is: *why have chemical biology approaches to study Kcr still not been developed?*

To promote and accelerate the in‐depth understanding of Kcr, unique and selective chemical tools and techniques must be developed. Before diving into what is available and known to study Kcr, we will give a few more insights into the enzymatic regulation of this PTM, raising other fundamental scientific questions.

## 2 Enzymatic Regulation of Lys Crotonylation (Kcr)

As described above, Kcr plays an important role in health and disease, therefore its investigation on a biomolecular level could have large impact on the development of new treatment options. From the moment of discovery researchers have been trying, with scarce success, to unravel how Kcr works mechanistically,^[57]^ which enzymes are able to catalyse the addition of the crotonyl group on the lysine residue, which proteins specifically recognise Kcr, and which can remove the crotonyl group; in other words: which are the *writers*, *readers*, and *erasers* of crotonylation, respectively? To date, only YEATS and DPF domain proteins have been characterised as H‐Kcr‐preferential *readers*; on the contrary, Kcr‐selective *writer* and *eraser* enzymes have not been discovered yet.^[59]^

### 2.1 Writers

**Figure 3:**
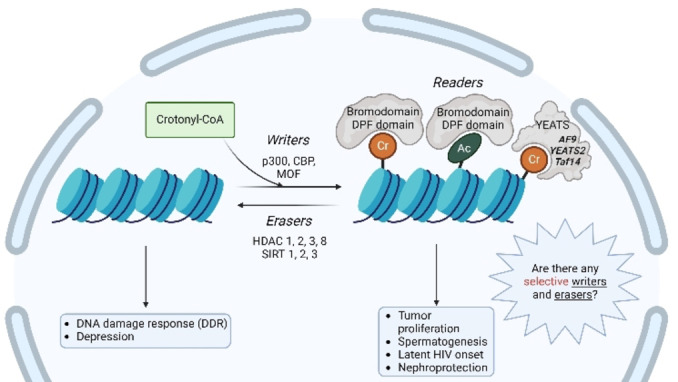
Overview of known *writers*, *readers*, and *erasers* for Kcr. The only selective enzymes identified for Kcr are *readers* containing a YEATS domain, raising the question whether there are also selective *writers* and *erasers* for this PTM.

In 2015, Sabari *et al*. identified the first *writer* enzyme for Kcr; they showed that p300/CBP, a previously known histone acetyltransferase (HAT) also exhibited histone crotonyl transferase (HCT) activity.^[35]^ The authors observed a decrease in the global levels of H3K18cr and H3K18ac when knocking down either p300 or CBP. Interestingly, *in vitro* experiments showed that H3K18 exhibits a larger affinity for crotonylation than for acetylation and that p300‐catalysed histone Kcr stimulates transcription to a greater degree than histone Kac.^[35]^ Up till now, p300 and CBP are still the major HCTs known in mammalian cells, however, they are not the only ones.^[60]^ MOF, also known as lysine acetyltransferase 8 (KAT8), is another HAT that has been shown able to catalyse histone Kcr at multiple sites.^[60]^ Interestingly, Peng *et al*. found out that the acetyl‐transferase p300 cannot catalyse p53 crotonylation *in vitro*, but HEK293T cell lysates could serve as a source of unidentified crotonyl transferases.^[52]^ All the *writers* identified for Kcr are shared with Kac and the selective transferase activity of the enzymes seems to be only correlated to the intracellular levels of the respective coenzyme substrates.^[35]^ Considering the lack of studies at the molecular level, it might be possible that selective *writers* for Kcr have not been identified yet (Figure 3).

### 2.2 Readers

**Figure 4:**
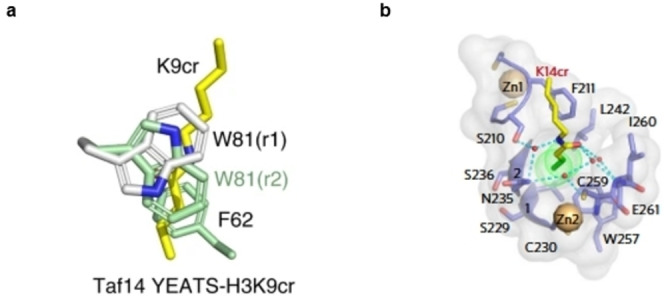
A). Crystal structure of H3K9cr in the Taf14 binding pocket, with K9cr indicated as yellow stick, showing triple *π*‐stacking, with the crotonyl group being sandwiched between W81 and F62;^[64]^ B). Composition of the K14cr‐binding β2 pocket of MOZ, with K14cr shown as yellow stick with its extended two‐hydrocarbon group highlighted in green. Images adapted from corresponding publications (PDB ID: 5IOK).^[66]^

*Readers* are proteins that can *‘read’* a specific PTM, recognising the functional group, and consequently sending a signal downstream.^[61]^ Three classes of proteins have been identified as *readers* selective for crotonylation: YEATS, bromodomain, and double PHD finger (DPF) domain. Out of these three, YEATS domain *readers* have the highest affinity towards Kcr, with YEATS2, AF9, and Taf14 being identified as the first class of selective Kcr *readers*.[^59^, ^62^, ^63^] The binding pocket of YEATS proteins contains an ‘end‐open’ feature, raising the hypothesis that longer and more rigid structures like Kcr are better recognised than Kac. This was confirmed by Li *et al*. when they showed that the crotonyl group fits perfectly in the ‘end‐open’ conformation of the AF9 YEATS domain.^[63]^ Within the binding pocket, the crotonyl group is sandwiched between the two aromatic residues of tryptophan and phenylalanine, due to π‐bond interactions.^[63]^ The same aromatic‐π‐stacking is reported for the YEATS2 and Taf14 domains, explaining the selectivity of these enzymes for Kcr (Figure 4a).[^59^, ^64^] Bromodomains, on the other hand, lack this Kcr preference. The affinity of TAF1 bromodomain to Kcr is lower compared to Kac, possibly due to a smaller binding pocket compared to the YEATS domain *readers*. Flynn *et al*. screened nearly the complete human bromodomain family for binding to different lysine acylations and found that whereas almost all bromodomains are capable of binding to shorter acylations, only TAF1 second bromodomain showed binding to crotonyl marks.^[65]^ As the third class of Kcr *reader*s, the PDF domains of human MOZ and DPF2 contain a larger binding pocket.^[66]^ Xiong *et al*. showed that MOZ and DPF2 bind to a wide range of histone lysine acylation, with the strongest preference for Kcr.^[66]^ This preference is a result of the hydrophobic interactions with the crotonyl group in the ‘dead‐end’ of the pocket and an amide‐sensing hydrogen bonding network (Figure 4b).^[66]^

### 2.3 Erasers

There are only two classes of enzymes known to be capable of erasing crotonyl groups from lysine residues: *histone deacetylase (HDAC) class I*, which are Zn^2+^‐dependent deacetylases and *sirtuins* (HDAC class III), which are NAD^+^‐dependent deacetylases.[^26^, ^67^] In 2012, Madsen and Olsen showed that HDAC3 exhibits histone decrotonylase (HDCR) activities *in vitro*.^[68]^ Since then, also the other members of the HDAC class I family, HDAC1, HDAC2, HDAC3, and HDAC8, have been shown to have both HDAC and HDCR activity.^[69]^ Moreover, Bao *et al*. found that SIRT1, SIRT2, and SIRT3 also have the ability to catalyse the hydrolysis of histone Kcr.[^70^, ^71^] A global crotonylation profiling by Xu *et al*. showed that knocking down HDAC1 and HDAC3 caused an increase in Kcr levels and that HDAC1, HDAC3, and SIRT2 can erase the crotonyl group on non‐histone proteins.^[42]^

As previously stated, no selective *writers* or *erasers* for Kcr have been found yet and also among non‐selective enzymes, not many have been associated to Kcr. It could be of interest to look at enzymes known to catalyse the addition or removal of similar lysine acylations and evaluate their crotonyl transferase or deacetylase activity; for example, it has been found that lysine butyrylation (Kbu), shares some *writers* and *readers* with Kcr.[^65^, ^72^] However, in the case of Kbu, selective *erasers* have been identified, with SIRT7 showing most activity.^[73]^ Such similarities raise the question why no selective *writers* and *erasers* are known for Kcr. A possible explanation for the lack of selective *erasers* might be that these are not part of either the HDAC or SIRT family; most studies aiming to identify cellular HDCR activity focus only on these two families, considering the known deacylase activities of these enzymes.^[69]^ Development of new methods for a broader investigation of Kcr *writers*, *readers*, and *erasers* has the potential to identify selective enzymes for Kcr, further broadening the understanding of Kcr on a cellular level and furnishing new therapeutic target proteins.

## 3 Origins of Crotonate: Metabolic Pathways and Sources

To crotonylate proteins, *writer* enzymes require crotonyl‐CoA as cofactor.^[35]^ Crotonyl‐CoA is not an endogenous molecule that exists freely in the human body, however, through cellular metabolic pathways crotonyl‐CoA can be generated, as it is for all acyl‐CoAs.^[74]^ The concentration of individual acyl‐CoAs was found to dictate the stoichiometry of acylations on chromatin and can change based on metabolic signals, such as those induced by diets, nutrition, and supplementation.[^19^, ^35^, ^75^] A variety of metabolites are produced by the gut microbiome through dietary fermentation, with short‐chain fatty acids (SCFA) being a significant portion.^[76]^ SCFAs are carboxylic acids that have an aliphatic tail consisting of two to six carbons. Although the body can naturally create SCFA in the liver, colonic bacteria are the main source of these compounds.^[76]^ About 95 % of the SCFA in the body is found to be acetate (C2), propionate (C3), and butyrate (C4).^[77]^ Each SCFA is present in the gut in millimolar concentrations, and several factors determine both the absolute molar quantities and their relative proportional ratios.^[76]^ These include the makeup of the microbiota, the fermentation site, and – possibly most important – the diet.^[78]^

**Figure 5:**
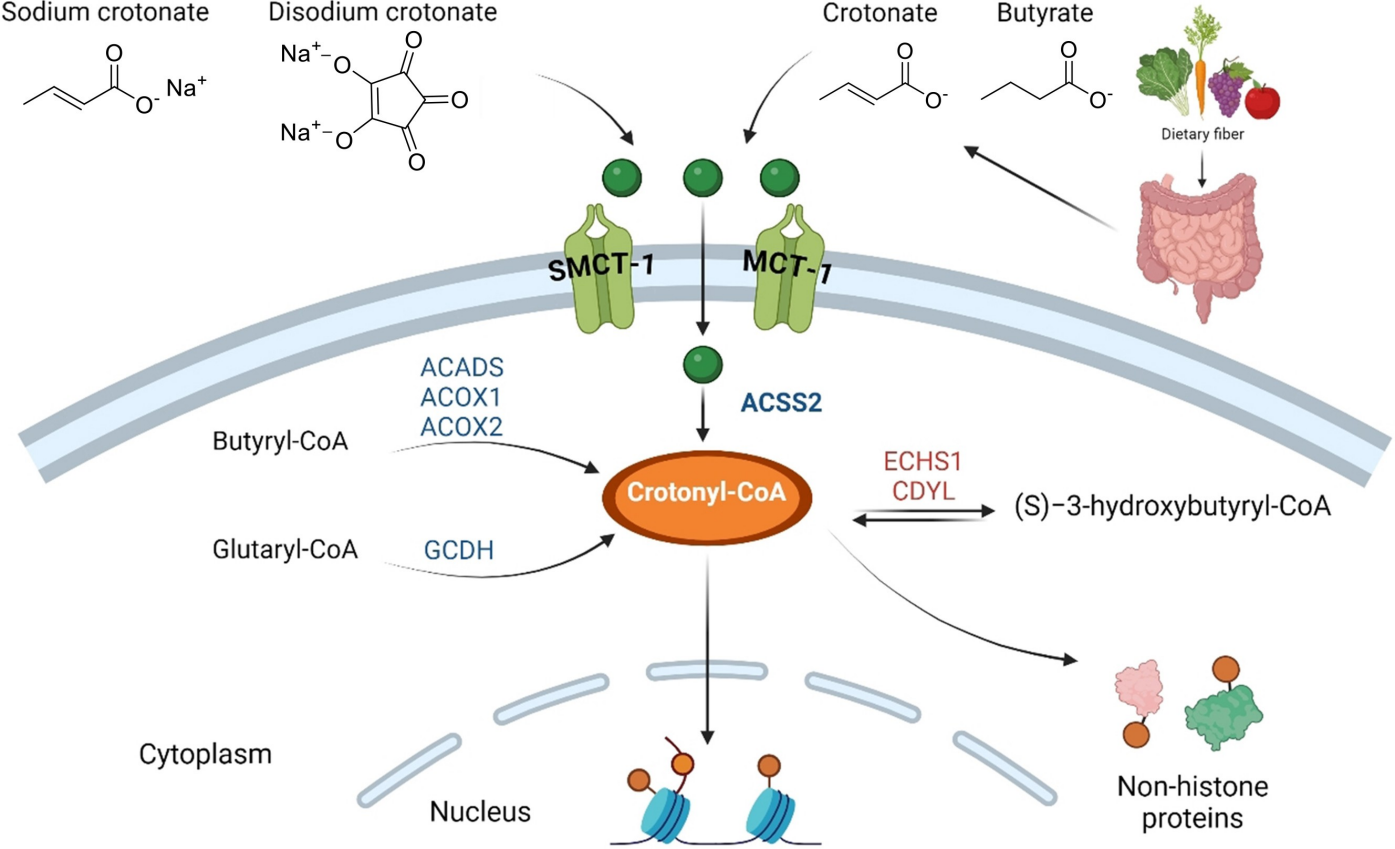
Possible mechanism of crotonylation. Intestinal microbiome produces the short‐chain fatty acids crotonate and butyrate through dietary fermentation. Crotonic acid enters the cytoplasm through diffusion or MCT‐1 vectors, while crotonate enters through SMCT‐1 vectors. Butyrate enters through both SMCT‐1 and MCT‐1. They are both converted into crotonyl‐CoA through ACSS2. Butyryl‐CoA, glutaryl‐CoA, and (*S*)−3‐hydroxybutyryl‐CoA can all be converted into crotonyl‐CoA. Enzymes in blue are the ones promoting crotonyl‐CoA production, and in red are the enzymes promoting hydrolysis of crotonyl‐CoA. Sodium crotonate and disodium crotonate are analogues of crotonic acid commonly used in experiments to upregulate crotonylation. After conversion into crotonyl‐CoA, both non‐histone proteins in the cytoplasm and histone proteins in the nucleus can be crotonylated.

Along with its analogues, crotonic acid enters the cell where it is converted into crotonyl‐CoA, a main substrate for Kcr (Figure 5).^[43]^ However, the metabolism of crotonic acid is not well explored and in need of more research. It is speculated that the main source of crotonyl‐CoA is the gut microbiome, as removal of the gut microbiota in antibiotic‐treated animals reduced serum and luminal SCFA levels while also lowering histone crotonylation.^[79]^ Both crotonic acid and analogues, such as disodium crotonate and sodium crotonate, have been used in cellular experiments to upregulate Kcr levels.[^5^, ^43^, ^80^]

Crotonic acid enters the cell through free diffusion or MCT‐1 vectors, and crotonate enters cells through SMCT‐1 vectors (Figure 5).^[43]^ Both are converted into crotonyl‐CoA through the metabolic enzyme acyl‐CoA synthetase 2 (ACSS2 or AceCS1), which is also known to produce acetyl‐CoA from acetate.^[81]^ Although ACSS2 knockdown has shown to lead to a significant decrease in Kcr expression, *in vitro* studies have not directly proven that ACSS2 can synthesise crotonyl‐CoA from crotonate. An indirect effect is also hypothesised, as the amount of acyl‐CoA is reduced after ACSS2 knockdown.[^35^, ^82^] Acetyl‐CoA can be used for fatty acid synthesis and therefore β‐oxidation of fatty acids. This is indeed an alternative route of crotonyl‐CoA biosynthesis.^[81]^ Fatty acid molecules are broken down by mitochondrial β‐oxidation forming acetyl‐CoA, leading to the generation of a crotonyl‐CoA intermediate upon oxidation of butyryl‐CoA, catalysed by acyl‐CoA dehydrogenase (Figure 5).[^43^, ^81^] Additionally, crotonyl‐CoA may be generated upon oxidative decarboxylation of glutaryl‐CoA, catalysed by glutaryl‐CoA dehydrogenase (GCDH) (Figure 5).^[43]^ The next step in this pathway is hydration of crotonyl‐CoA to 3‐hydroxybutyryl‐CoA that is catalysed by short‐chain enoyl‐CoA hydratase 1 (ECHS1) (Figure 5).^[81]^ However, it is hypothesised that some crotonyl‐CoA escapes degradation, leaking from the mitochondria thus contributing to histone crotonylation. Consistent with this are the findings that histone Kcr is mediated by fatty acid β‐oxidation pathway in yeast.^[83]^ Furthermore, this pathway has recently sparked interest as glioblastoma stem cells have been found to reprogram lysine catabolism to aid their proliferation.^[84]^ Namely they noticed a GCDH overexpression together with downregulation of ECHS1, which led to an increased pool of crotonyl‐CoA through reprogrammed lysine catabolism. Two additional fatty acid oxidation enzymes, ACADS and ACOX3, were identified as key crotonyl‐CoA producers during endoderm differentiation, with their mechanisms unfortunately being unclear.[^85^, ^86^] The SCFA butyrate, along with sodium butyrate, has also been shown to promote histone crotonylation in various studies.[^79^, ^87^, ^88^] For example, butyrate produced by gut bacteria was shown to increase histone crotonylation in colon epithelial cells and in another study butyrate reduced hypoxia‐induced brain damage by regulating crotonylation of histone and non‐histone proteins.[^79^, ^87^] Whereas crotonate was able to reduce invasive growth and immune escape of *Candida albicans* by mediating hyphal gene expression.^[89]^ Although it has been hypothesised that histone Kcr connects chromatin to the gut microbiota via SCFAs and HDACs, a lot is still to be learned about the role of crotonylation in the microbiome and host‐microbe interactions.[^79^, ^89^] Not only is there a lack of studies on crotonate production by bacteria, but also the dependence of crotonate levels on certain foods or diets. Currently, crotonate and butyrate seem to be the major direct sources of crotonyl‐CoA; it is therefore necessary to deduce whether other pathways, such as breakdown of other essential amino acids and fatty acid β‐oxidation are significant sources of crotonyl‐CoA. Furthermore, understanding the source of crotonylation along with the biological metabolism will allow better understanding of not only Kcr, but also its relationship with the gut microbiota, allowing for new potential treatments, as this has already been implicated in dietary interventions.^[84]^

## 4 Methods to Study Kcr Proteins and its Partners

**Figure 6:**
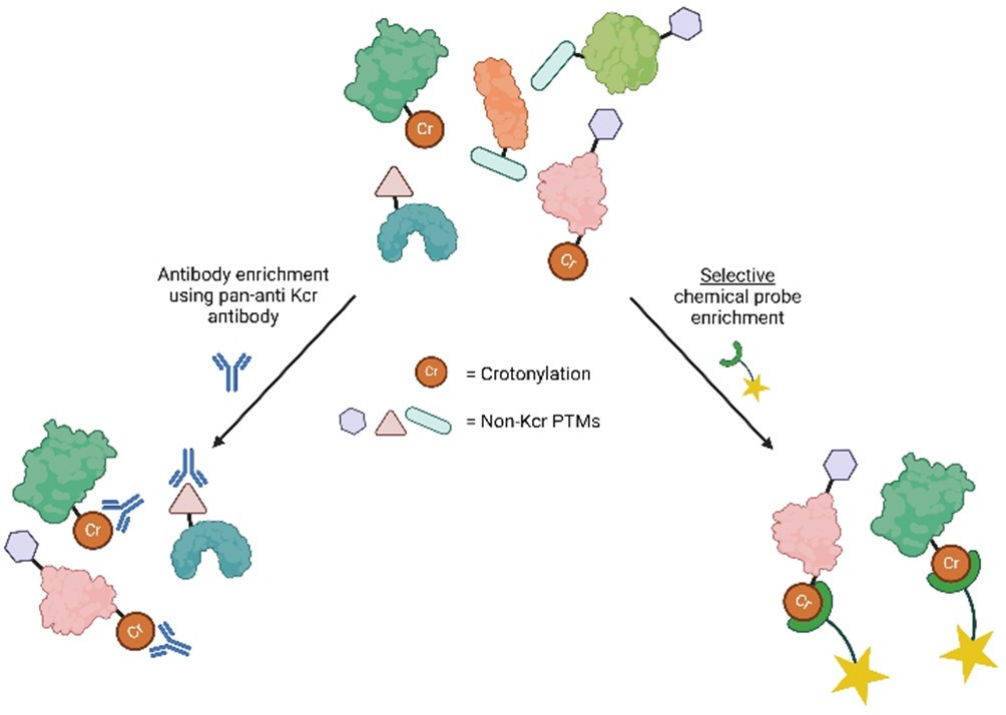
Schematic representation of the non‐selective enrichment using the anti‐Kcr antibody compared to chemical probe enrichment.

Scientists’ ability to identify and map PTMs has improved dramatically due to the huge advancements in sensitivity, speed, accuracy, and resolution of MS methods.^[90]^ However, proteomics analysis without an enrichment step prior to MS analysis is not sufficient for the detection and characterisation of low abundance PTMs. Several enrichment strategies can be used to circumvent this issue.[^90^, ^91^] The most commonly used strategy for the identification of PTMs employs a selective PTM antibody. This antibody was combined with MS for the first time almost three decades ago.^[92]^ Immunoaffinity purification using a pan‐PTM antibody coupled to LC–MS/MS has been successful for the global analysis of several PTMs, including Kac,[^93^, ^94^] arginine methylation,^[95]^ tyrosine nitration,^[96]^ and tyrosine phosphorylation.[^97^, ^98^] Concerning Kcr, Tan *et al*. developed the first pan‐antibody against this PTM; the antibody could recognise peptide libraries bearing Kcr moieties and was subsequently used for western blotting analysis to study protein crotonylation.^[14]^ Both Wei *et al*. and Xu *et al*. used the same pan‐anti Kcr antibody enrichment coupled to LC–MS/MS to identify non‐histone proteins. Interestingly, using the same technique, Wei *et al*. identified 1185 Kcr sites on 453 non‐histone proteins after treatment with sodium crotonate, whereas Xu *et al*. identified 2696 Kcr sites on 1024 non‐histone proteins.[^41^, ^42^] These differences were possibly due to the lack of high selectivity of the anti‐Kcr antibody, which was already reported by Tan *et al*. when the antibody was used for the first time (Figure 6).^[14]^ Afterwards more and more groups have reported cross‐reactivity of the antibody with other acylations, raising the question how reliable and accurate the currently reported crotonylome descriptions actually are.[^99^, ^100^]

This is not the first time the question has been raised whether immunoaffinity purification is the best enrichment method. The most difficult part of antibody‐based enrichment is the need for a selective and sensitive anti‐PTM antibody. Most PTMs, especially acylations, are relatively small and structurally similar, making is difficult to generate pan‐PTM selective antibodies, which recognise a certain PTM independent of its surrounding sequence.[^91^, ^101^] Moreover, epitope occlusion, the situation in which adjacent modifications prevent antibody recognition, leads to reduced selectivity in immunoaffinity assays.^[102]^ These difficulties are also observed for Kcr, as the pan‐anti Kcr antibody recognies other acylations, like Kbu, in addition to Kcr proteins.[^14^, ^41^]

An efficient enrichment strategy, that circumvents the use of selective antibodies, is based on the use of chemical probes.^[100]^ Chemical probes require a tag, for example biotin, for enrichment and a reactive group that can selectively bind the target protein or modification. A prerequisite for this is that the protein of interest contains a functional group that can selectively and covalently react with the probe. When incubated with a biological sample, the selective chemical probe would first enable the selective labelling of the PTM, then allowing the selection only of the labelled proteins, in the so‐called enrichment step. The use of a covalent chemical probe thus represents a more versatile, cost‐effective, and sensitive tool for the identification of low abundance PTMs (Figure 6). This approach has been successfully applied for the identification of several protein PTMs.^[103]^ For example, in 2022 Ray *et al*. labelled and characterised glyoxal modified histones in a quantitative proteomics approach using an aniline‐derived probe that selectively reacted with glyoxal.^[104]^ In the same year, Wang *et al*. published new methodology to study arginine demethylation, based on a steric effect‐based chemical enrichment strategy.^[105]^

**Figure 7:**
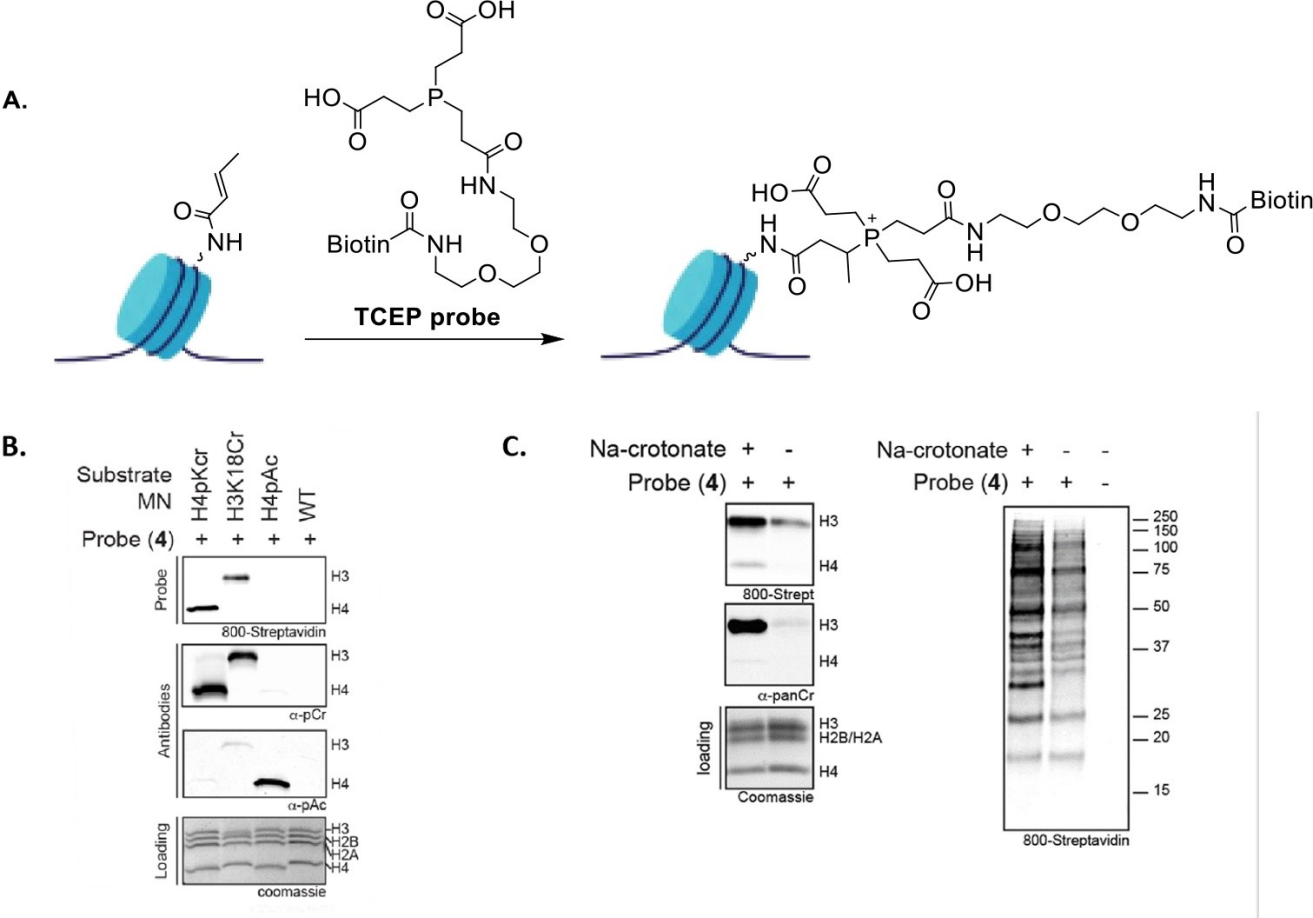
A). Covalent capture of crotonylated histones by the biotinylated TCEP probe, the first chemical probe for Kcr; B). Polycrotonylated (H4pKcr), monocrotonylated (H3K18cr), polyacetylated (H4pAc), and unmodified (WT) nucleosomes were incubated with the TCEP probe and analysed by western‐blot. The same nucleosomes were analysed by western blotting employing the pan‐anti Kcr and pan‐anti Kac antibody; C). Western blot analysis showing Kcr levels of either acid extracted histones (left panel) or nuclear extract (right panel) from HEK93 cells, either grown in the presence or absence of sodium crotonate. Adapted from.^[106]^

In 2018, Bos and Muir introduced the first chemical probe for crotonylated proteins.^[106]^ The Kcr‐probe is based on a water‐soluble phosphine warhead containing a pendent carboxylic acid group, which in presence of the crotonyl mark generates β‐phosphonium species (Figure 7a). Tris(2‐carboxyethyl)phosphine (TCEP) was found to be highly reactive towards crotonylated model peptides so they built a TCEP‐based chemical probe where a biotin moiety was coupled to one of TCEP carboxylic groups enabling affinity enrichment and streptavidin‐based blotting analysis of crotonylated proteins.^[106]^ Histone H3 with a Kcr group at position 18 (H3K18cr) was used as an initial test to investigate the reactivity of the TCEP probe and upon incubation, high biotin incorporation was detected in both H3 (H3K18cr) and H4 (H4pCr). (Figure 7b). No biotin was incorporated into nucleosomes with multiple preinstalled Kac marks (H4pAc) or into unmodified nucleosomes (WT) (Figure 7b). Furthermore, the probe was used to selectively target crotonylated histone proteins in a cell lysate. HEK293 mammalian cell lines were grown in presence of sodium crotonate, which is known to upregulate crotonyl‐CoA levels.After treatment with the TCEP probe, endogenous crotonylated histone proteins were indeed enriched (Figure 7c).

Although of great impact and novelty, this work shows some grey areas. For example, it is not exactly clear why the presence of the two free carboxylic groups is fundamental for the probe reactivity; the authors hypothesised that the negatively charged carboxylate groups stabilise the formed phosphonium cation through electrostatic interactions. However, they also speculated that hydrogen bonding interactions or the involvement of a transient cyclic phosphorane species could play a role in the reaction mechanism.^[106]^ Unfortunately, the correct mechanism remains unknown as no further investigation was reported. Surprisingly, the probe was never used in proteomics analysis raising the question whether it would actually be compatible with a standard chemoproteomics workflow, as phosphine warheads are generally not selective, synthetically demanding, and have poor chemical tractability. Despite these limitations, the study provides clear evidence of the relevance of a selective chemical probe in investigating protein crotonylation within a complex proteome.

An additional important factor for the validation and characterisation of identified Kcr proteins is the ability to synthesise Kcr proteins. Synthesising proteins with defined PTMs can be challenging as PTMs are dynamic and can undergo changes under several conditions, resulting in heterogeneously crotonylated proteins when enzymatic synthesis is applied. For a long time, semi‐synthesis was the best method to synthesise proteins with defined PTMs, however, this can be a very time consuming and complex method.[^106^, ^107^, ^108^] The development of genetic code expansion using amber stop‐codon suppression to incorporate modified amino acids directly into target proteins in a desired position really boosted the synthesis of homogeneously PTMs‐modified proteins.[^109^, ^110^, ^111^] In 2012, Kim *et al*. synthesised *N* ^*ϵ*^‐crotonyllysine and site specifically incorporated it into histones using genetic code expansion for the first time.^[112]^ This method is now commonly used for the synthesis of crotonylated proteins and offers a perfect background for the synthesis of different Kcr‐modified proteins.

**Figure 8:**
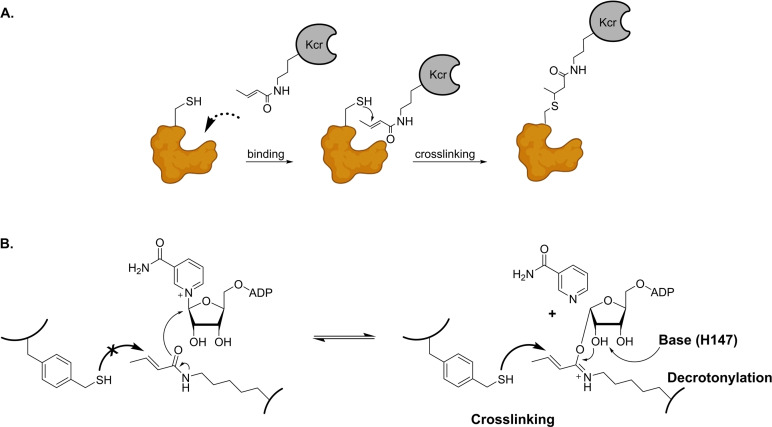
A). Covalent capture of Kcr‐interacting proteins via proximity‐induced cross‐linking; B). Schematic representation of the reversible formation of *O*‐alkylamidate and its effect on cross‐linking and decrotonylation.^[113]^

On a different approach compared to the selective chemical TCEP probe, Ji *et al*. developed the first protein‐based covalent binder for selective recognition of crotonylated proteins (Figure 8a).^[113]^ This strategy is based on proximity‐induced cross‐linking, which has drawn a big attention in the study of protein‐protein interactions. The authors used CobB, a NAD^+^‐dependent sirtuin‐derived protein as binding target for Kcr proteins, because of its known decrotonylase activity.^[114]^ They demonstrated that a thiol handle close to the binding site of crotonylated interacting proteins can react with *O*‐alkylamidate, the adduct of NAD^+^ and Kcr (Figure 8b). Crotonylated ubiquitin Kcr‐S1 was used as a substrate to evaluate the cross‐linking reactivity of CobB, and non‐canonical amino acids with thiol‐methyl groups at hydrophobic positions F60, W67, and Y92 of CobB were incorporation due to the proximity at the binding site of Kcr‐S1. Following the mixture of the reactants, western blot analysis showed a clear formation of cross‐linked products for all three mutants, and cross‐linking was found more effective in presence of the sirtuin cofactor NAD^+^. Importantly, the Kcr‐binder showed selective cross‐linking reactivity against Krc‐S1, in presence of acetylated substrate Kac‐S1. In the presence of NAD+, the fluorescently labelled protein‐based Kcr‐binder successfully recognised crotonylated targets in HeLa cells that were treated with sodium crotonate, indicating the importance of this cross‐linking strategy in targeting crotonylation in fixed cells. A drawback of this strategy is that only Kcr proteins that are recognised by CobB can be identified in this way.

Another commonly used method to identify PTM‐containing proteins is *in vitro* metabolic labelling.^[91]^ In this method a small, bio‐orthogonal tag, often an azide, is conjugated to the PTM precursor and metabolically incorporated. The resulting chemically labelled PTM protein can be conjugated to an affinity linker, for example an alkyne containing biotin, for successive enrichment. The success of this approach highly depends on enzyme recognition, since the small bio‐orthogonal tag creates an artificial system, which changes the nature of the PTM, potentially impacting its protein incorporation efficiency. Metabolic labelling was successfully used for the identification of several acylated proteins.[^115^, ^116^] Interestingly, to our knowledge this method has not yet been applied for the identification of Kcr proteins and might form a complementary strategy to the existing ones.

Apart from the direct identification of Kcr proteins, the discovery of selective Kcr‐interacting proteins, like *writers*, *readers*, and *erasers*, would also provide valuable information in the understanding of its role and regulation mechanism in cells. Moreover, such enzymes form potential therapeutic targets for modulating Kcr. There are some probes developed for Kac‐interacting proteins, and thus also for some Kcr *writers*, *readers*, and *erasers* as some of these proteins recognise both PTMs. For example, Humphreys *et al*. developed a chemical probe for the p300/CREB binding protein association factor^[117]^ and Li *et al*. developed chemical probes for YEATS domains by targeting the π‐π‐π staking.^[118]^ Recently, Chen *et al*. published a new strategy for the discovery of new potent epigenetic *reader* protein inhibitors.^[119]^ This approach is based on a phage display library in which each displayed peptide contains a genetically encoded non‐canonical amino acid with an epigenetic mark that is known to bind to the *reader* protein of interest. In their study, Chen and coworkers incorporated *N^ϵ^‐butyryl‐lysine*, which is known to bind to ENL YEATS. After several enrichment rounds, they obtained a pool of potent inhibitors, which they further optimised. This seems to be a promising strategy for the identification of Kcr‐interacting inhibitors, however, in order to apply this method, it is crucial to first identify selective Kcr *writers*, *readers*, and *erasers*.

**Figure 9:**
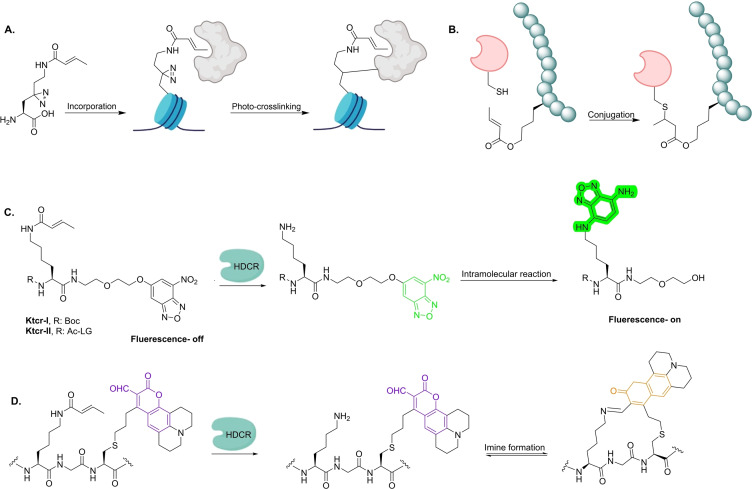
A). Photo‐cross‐linking strategy towards the detection of Kcr‐interacting targets.^[123]^ B). Ester‐derived crotonyl‐mimic probe for capturing of H3K27cr interacting proteins.^[124]^ C).^[125]^ and D). Mechanistic approach for the identification of decrotonylases using fluorescent Kcr‐peptide probes.^[126]^

A valuable method for the identification of Kcr‐interacting proteins is the use of photoaffinity probes.[^120^, ^121^, ^122^] This method has recently been applied by Xie *et al*. when they reported the development of a genetically encoded biosensor for the recognition of enzymatic machinery for histone lysine crotonylation.^[123]^ Site‐specific incorporation of diazirine‐containing amino acids into a protein of interest permits the conversion of non‐covalent interactions into covalent linkages, which is important in the studies of protein‐protein interactions. In this work, crotonylated lysine bearding a diazirine group (K*Cr) was genetically encoded at position 79 of recombinant H3 protein in *E. coli*, enabling the capturing of interacting proteins (Figure 9a). H3 K*79cr was treated with SIRT3 which is a known histone decrotonylase, and upon UV irradiation, western blotting showed a clear formation of cross‐linked product. This strategy was next performed in HEK293T mammalian cells expressing the H3K*79cr protein. After irradiation, western blotting analysis showed new distinct photo‐cross‐linked bands. Thus, this genetic code expansion strategy can be used as a useful tool to identify more Kcr protein partners, such as *readers* and *erasers*.

Peptide‐based probes bearing crotonylated lysine have been extensively used to study the Kcr‐specific interactome. Guo *et al*. developed a new lysine crotonyl‐mimic peptide probe which could identify histone Kcr‐interacting proteins via proximity‐cysteine bioconjugation (Figure 9b).^[124]^ The amide group at the crotonyl moiety of the peptide probe was replaced by an ester group, providing a more reactive Michael acceptor. Based on this approach, the first 13‐mer peptide probe H3g27Cr was constructed as a H3K27cr modification probe, bearing an ester‐modified crotonyl mark at position 27. After demonstrating that H3g27cr maintains the recognition with the H3K27cr antibody, its ability to capture H3K27cr‐interacting proteins was evaluated. Incubation of HDAC1, a known protein decrotonylase, with the H3g27cr probe led to a clear formation of conjugation product. Moreover, biotinylated H3g27cr was incubated with a A549 cell lysate for pull‐down assay, and the probe enriched 89 proteins. Interestingly, signal transducer and activator 3 (STAT3) was one of the enriched proteins which had only been reported as a possible interacting protein with H3K27cr. To further validate the unknown H3K27cr‐STAT3 interaction, a STAT3‐HEK‐293 cell lysate was incubated with the peptide probe leading to successful pull‐down of STAT3 protein.

Xie *et al*. developed the first single‐step activity‐based fluorescent probes for detection of decrotonylation activity (Figure 9c).^[125]^ The formation of the free amine group following decrotonylation, leads to a rapid intramolecular rearrangement of the peptide probe yielding a highly fluorescent product (Figure 9c). The two synthesised probes KTcr‐I and KTcr‐II allowed the real‐time monitoring of decrotonylation activities of SIRT2 and HDAC3, respectively, with generation of a strong fluorescent signal following incubation. Similarly, Rooker *et al*. reported a single‐reagent fluorescent crotonylated peptide probe for identification and evaluation of decrotonylases using fluorescence‐based assays.^[126]^ Following enzymatic decrotonylation, the peptide probe undergoes a spontaneous imine formation which results in a remarkable shift in the absorption spectrum. Incubation of the probe with the recombinant HDAC3/NCOR1 complex resulted in a significant increase of absorption at λ=565 nm, proving the strong decrotonylation activity of the complex.

Overall, many new methods to study Kcr have recently been discovered, especially for the identification of Kcr‐interacting proteins. However, selective methods for the identification of Kcr proteins themselves are still lacking; most of the research that has been conducted is based on the pan‐Kcr antibody, even though its reliability remains questionable. The development of novel chemoproteomics approaches combined with recent advancements in protein ligation strategies will push the discovery of Kcr proteins and Kcr‐interacting enzymes, leading to a more in‐depth understanding of this PTM.

## 5 Summary and Outlook

Protein post‐translational modifications (PTMs) play a crucial role in various cellular processes. To date, over 400 PTMs have been identified, with dysregulation linked to diseases like cancer and cardiovascular disease. Advanced proteomic techniques have uncovered new PTMs, including short‐chain (SC) lysine acylations. Among these, lysine crotonylation (Kcr) is particularly noteworthy. Kcr is a critical PTM with significant roles in health and disease, presenting a promising avenue for therapeutic development.

The study of Kcr is advancing, yet significant gaps remain, particularly in understanding its regulation and identifying Kcr‐interacting proteins. To date, only YEATS and DPF domain proteins have been identified as preferential *readers* of Kcr, with p300/CBP/MOF and HDAC known as non‐selective *writers* and *erasers*, respectively.

The absence of selective Kcr *writers* and *erasers* highlights a promising area for further investigation. Broadening the search beyond the HDAC families could for example represent an interesting approach to tackle this problem.

Another interesting unanswered question relates to the metabolism of crotonate. As described above, crotonyl‐CoA production involves several pathways, including the β‐oxidation of fatty acids and the metabolism of essential amino acids. Enzymes such as acyl‐CoA synthetase 2 (ACCS2) and glutaryl‐CoA dehydrogenase (GCDH) play critical roles in these processes. However, the exact mechanisms and contributions of these pathways to crotonyl‐CoA levels are not fully understood.

Emerging evidence suggests that gut microbiota significantly influence crotonyl‐CoA production; studies have shown that the removal of gut microbiota in animals reduces histone crotonylation, highlighting the microbiome's role in this process. Future research could then focus on the elucidation of the detailed mechanisms of crotonyl‐CoA biosynthesis, the role of different metabolic pathways, and the influence of diet and microbiota on Kcr. Additionally, dietary interventions affecting SCFA levels could potentially modulate Kcr and its biological effects.

Finally, although immunoaffinity purification using pan‐PTM antibodies, has facilitated the identification of Kcr sites, issues with selectivity and cross‐reactivity limit the reliability of the currently published works. None of the reported descriptions of the crotonylome overlap, even though same methods are used.

Chemical probe‐based enrichment offers a promising alternative, allowing selective and sensitive detection of PTMs. For instance, phosphine‐based chemical probes have been developed for Kcr, enabling the enrichment and analysis of crotonylated proteins. However, these probes seem to have practical limitation to proceed with further analyses, leaving a huge gap in this area of investigation.

Despite these advances, the development of selective chemical tools for Kcr remains incomplete. Future research should focus on refining these methods to achieve more precise and comprehensive profiling of Kcr and its regulatory mechanisms.

## Conflict of Interests

The authors declare no conflict of interest.

## Biographical Information

*Marinda Westerveld graduated cum laude from College of Pharmaceutical Sciences (BSc) at Utrecht University in 2018. She did two internships while following the master's programme Drug Innovation at Utrecht University: one in the Wennekes group at Utrecht University focussing on ways to study the bacterial gut glycan metabolism and one in the Wennemers group at ETH Zurich where she worked on the functionalisation of peptide‐coated platinum nanoparticles. She started her PhD in 2023 under the supervision of Dr Rita Petracca at Utrecht University, where she is now working on the development of novel methods to study protein crotonylation*.

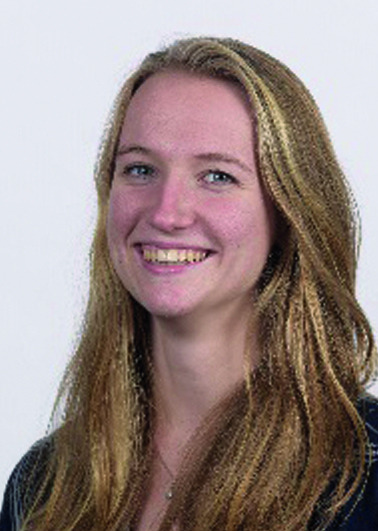

## Biographical Information

*Kosta Besermenji, born in Serbia, graduated Drug Development (BSc) at Utrecht University in 2023. During his studies he did a research project in Heck Lab on size exclusion chromatography of IgA under the supervision of Dr Amber Rolland and Dr Albert Bondt. Currently he is following Drug Innovation (MSc) at Utrecht University and is doing a project under the supervision of Dr Rita Petracca focused on screening new probes for the identification of the crotonyl group on various proteins*.

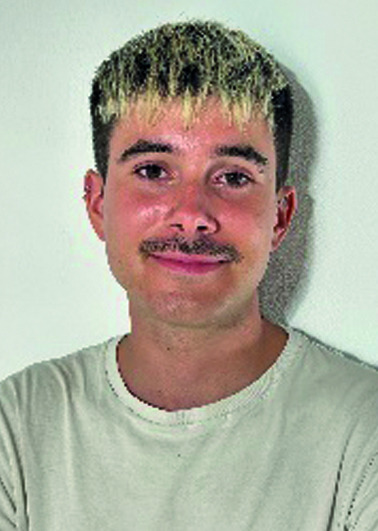

## Biographical Information

*David Aidukas is a MSc student at Utrecht University currently completing his Major project at Utrecht University, focusing on identifying possible new crotonylation* writers, reader*s, and* erasers*, guided by Dr Rita Petracca. Previously he has completed his Bachelors working on synthesis and optimisation of stereospecific carbohydrates. Afterwards, he has worked as a chemical technician at the lab, assisting in further optimisations of carbohydrate synthesis. David has extensive organic chemistry knowledge and currently is pursuing his interest in furthering his biological skills and understanding*.

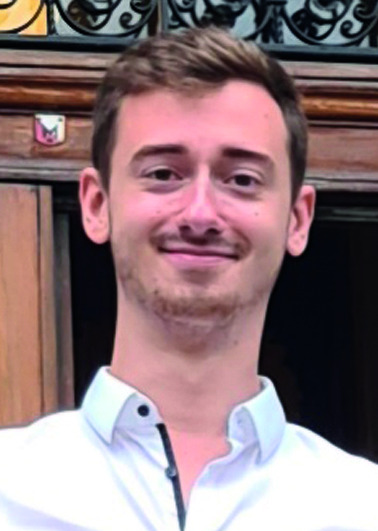

## Biographical Information

*Nikita Ostrovitsa graduated first‐in‐class in Chemistry (BSc) from the University of Patras in 2019. He then moved to London where he completed the MSc program in Drug Discovery and Development at Imperial College London. During this time, he joined the research group of Professor Ed Tate and worked on the design, synthesis, and biological evaluation of macrocyclic peptide inhibitors. In 2021 he started his PhD studies in Organic and Peptide Chemistry at Trinity College Dublin under the supervision of Professor Eoin Scanlan. His research focuses primary on the development of synthetic methodologies for peptide cyclisation and modifications*.

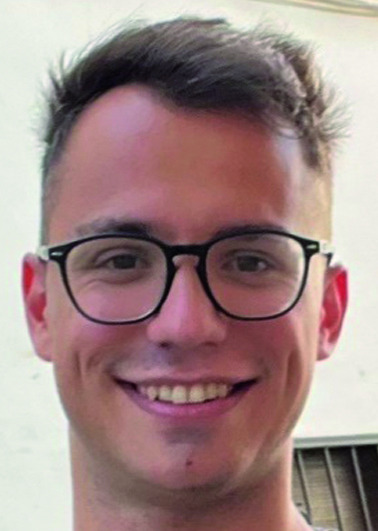

## Biographical Information

*Dr Rita Petracca graduated in Pharmaceutical Sciences and Technologies at the University of Salerno in 2011. She got a 5 year PhD fellowship at the Italian Institute of Technology in Genoa, and the PhD title in Drug Discovery in 2016, after spending 18 months in the lab of Prof Overkleeft in Leiden. She worked 2 years at Trinity College Dublin as postdoctoral fellow and 3 years at Imperial College London as a Marie Curie fellow. She recently joined Utrecht University as Assistant Professor in chemical biology to start her independent career. Rita's research focusses on the study of potein crotonylation*.